# Antiseptic Properties of Chloroform

**Published:** 1851-04

**Authors:** 

**Affiliations:** Constantinople


### MATERIA MEDICA AND THERAPEUTICS.

*Antiseptic properties of Chloroform. By M. Augend, of Constantinople.*—M. Augend has communicated to the French Academy of Sciences the following experiments, which establish a marked line of demarcation between ether and chloroform, and point out in the latter a remarkable property which has hitherto escaped the attention of chemists.

If three wide-mouthed, ground stoppered bottles are taken, and into one we put a few drops of ether, into another a few drops of chloroform, and leave the third in *statu quo*; then place in each a piece of beef, secure the stoppers, and leave them during summer weather, the following phenomena are observed:—The flesh, naturally of a reddish brown color, passes et once to a vermillion-red, under the influence of the vapor of chloroform mixed with air, whilst it undergoes no change in the ether. Such are the immediate effects, but at the end of a week the results are still more distinct. The flesh preserved in the air has changed its color slightly, that which is preserved in the ether has become brown, whilst that in the chloroform has acquired the appearance of boiled meat. On opening the bottles the meat in the air has become offensively putrid, and the same thing occurs in the presence of the ether, whilst there is no change in the flesh kept in the Chloroform, apart from the sweet taste and peculiar odor of the lattter.

These antiseptic properties of chloroform have furnished interesting results to M. Augend. He has found that 1-2000th part of it suffices to preserve a mass of muscular flesh. Not less remarkable is the facility with which the vapor permeates the densest tissues. Chloroform has this advantage over creasote, that it does not coagulate the albumen; nor is it on its part decomposed by muscular fibre.

The most apparent action of the chloroform, not only on muscular substance, but also on the fleshy pericarps of fruits and seeds, is an immediate contraction of the fibre or the parenchyma, which causes the watery juices to flow out on the bottom of the vessel in which the experiment is made.

The author reports a number of trials made on different animal substances, and modified in various ways, from which it results that the antiseptic properties of chloroform may be of use to naturalists and anatomists, who wish to preserve specimens for some time. He even thinks that it might be applied to the preservation of bodies in case of homicide, where these might require to be kept for sometime for medico-legal purposes.—*Gazette Medicale,* 15th November, 1850.

